# Distinct mutation profiles between primary bladder cancer and circulating tumor cells warrant the use of circulating tumors cells as cellular resource for mutation follow-up

**DOI:** 10.1186/s12885-020-07684-6

**Published:** 2020-12-07

**Authors:** Tae-Min Kim, Jin-seon Yoo, Hyong Woo Moon, Kyung Jae Hur, Jin Bong Choi, Sung-Hoo Hong, Ji Youl Lee, U-Syn Ha

**Affiliations:** 1 Department of Medical Informatics, College of Medicine, The Catholic University of Korea, Seoul, Republic of Korea; 2 Department of Urology, Seoul St. Mary’s Hospital, College of Medicine, The Catholic University of Korea, Seoul, Republic of Korea; 3 Department of Urology, Bucheon St. Mary’s Hospital, College of Medicine, The Catholic University of Korea, 327, Sosa-ro, Wonmi-gu, Gyeonggi-do, Republic of Korea; 4 Cancer Research Center, College of Medicine, The Catholic University of Korea, Seoul, Republic of Korea

**Keywords:** Bladder cancer, Circulating tumor cells, Mutation

## Abstract

**Background:**

While circulating tumor cells may serve as minimally invasive cancer markers for bladder cancers, the relationship between primary bladder cancers and circulating tumor cells in terms of somatic mutations is largely unknown. Genome sequencing of bladder tumor and circulating tumor cells is highlighted to identify the somatic mutations of primary bladder cancer.

**Methods:**

Bladder cancer tissue was collected by transurethral resection of the bladder and preserved by snap-freezing. Circulating tumor cells were Isolated from the blood obtained before treatment. We performed whole exome sequencing of 20 matched pairs of primary bladder cancers and circulating tumor cells to identify and compare somatic mutations of these two different genomic resources.

**Results:**

We observed that mutation abundances of primary bladder cancers and circulating tumor cells were highly variable. The mutation abundance was not significantly correlated between matched pairs. Of note, the mutation concordance between two resources was only 3–24% across 20 pairs examined, suggesting that the circulating tumor cell genomes of bladder cancer patients might be genetically distinct from primary bladder cancers. A relative enrichment of mutations belonging to APOBEC-related signature and a depletion of C-to-G transversions were observed for primary- and circulating tumor cells specific mutations, respectively, suggesting that distinct mutation forces might have been operative in respective lesions during carcinogenesis.

**Conclusions:**

The observed discrepancy of mutation abundance and low concordance level of mutations between genomes of primary bladder cancers and circulating tumor cells should be taken into account when evaluating clinical utility of circulating tumor cells for treatments and follow-up of bladder cancers.

**Trial registration:**

Patients were selected and registered retrospectively, and medical records were evaluated.

**Supplementary Information:**

The online version contains supplementary material available at 10.1186/s12885-020-07684-6.

## Background

Bladder cancer is a widespread and highly heterogeneous malignancy. Clinical outcome of this disease is poor because of its highly recurrent nature with frequent disease progression and treatment failure [1]. In clinical situation, patients with advanced bladder cancer are at high risk of disease recurrence or progression, with half of them having relapse after radical surgery. For example, 30–80% of bladder cancer patients with advanced disease below pT1 stages experienced disease recurrence and up to 45% of cases progressed to muscle invasion within 5 years [2–4]. The majority of relapses are distant metastasis and 10–15% of cases are already metastatic at diagnosis [5]. Consequently, bladder cancer is characterized with variable clinical outcomes requiring frequent follow-up and repeated treatments, making this disease obstinate and challengeable to overcome.

To overcome these hurdles, circulating tumor cells (CTCs) have been recently highlighted for “liquid biopsy” using the peripheral blood of patient as a resource of tumor cells. Ideally, CTCs can replace invasive tissue biopsies for serial monitoring of tumor characteristics and facilitate the revision of treatment or follow-up protocols [6].

High-throughput sequencing technologies have facilitated mutation screening of cancer genomes. Mutation profiles of individual cancer genomes have been traditionally used to prioritize cancer drivers (e.g., actionable alterations with available therapeutic options or predictive/prognostic markers). Moreover, an entire catalogue of mutations in given cancer genomes can be used as genetic markers to evaluate the extent of within-tumor heterogeneity between regional biopsies [7] or similarities between primary-vs.-metastatic lesions of given individuals [8]. In the case of CTC, it is challenging to estimate the extent as to how many mutations of primary tumors can be discovered by CTC which can be only evaluated by direct sequencing of primary tumor genomes and their matched CTC genomes. However, sequencing coverage to evaluate mutation-level concordance between primary tumors and CTC genomes has been largely limited due to the number of CTCs that is generally too few to obtain sufficient amount of DNA to perform exome-sequencing.

In this study, we isolated CTCs from 20 patients with urinary bladder cancers. For genomic analyses, we performed whole-exome sequencing of matched primary and CTC genomes. Somatic mutations were compared between primary tumor and CTC genomes to evaluate mutation-level concordance and identify the potential of CTCs as alternative cellular resources to identify somatic mutations of primary tumors.

## Methods

### Patient recruitment and collection of blood and tissue

A total of 20 patients were enrolled for this study from May 2016 to September 2017 under an Institutional Review Board–approved protocol of our institution, Seoul St. Mary’s Hospital, College of Medicine, the Catholic University of Korea and written informed consents were obtained (approval number: KC15TNSI0924). For each patient, bladder cancer tissue was collected by transurethral resection of the bladder (TURBT) and blood was collected before starting TURBT. A total of 15 cc of blood was divided into 5 ml for CTC enumeration and 10 ml for CTCs culture. All blood and tissue samples as well as medical data used were anonymous to ensure patient confidentiality. Primary tumor specimens were snap-frozen and histologically examined by pathologists after hematoxylin and eosin staining. Tissue blocks with tumor purity (> 70%) confirmed were used to extract genomic DNAs.

### Isolation of CTCs and enrichment process

**Fig. 1:**
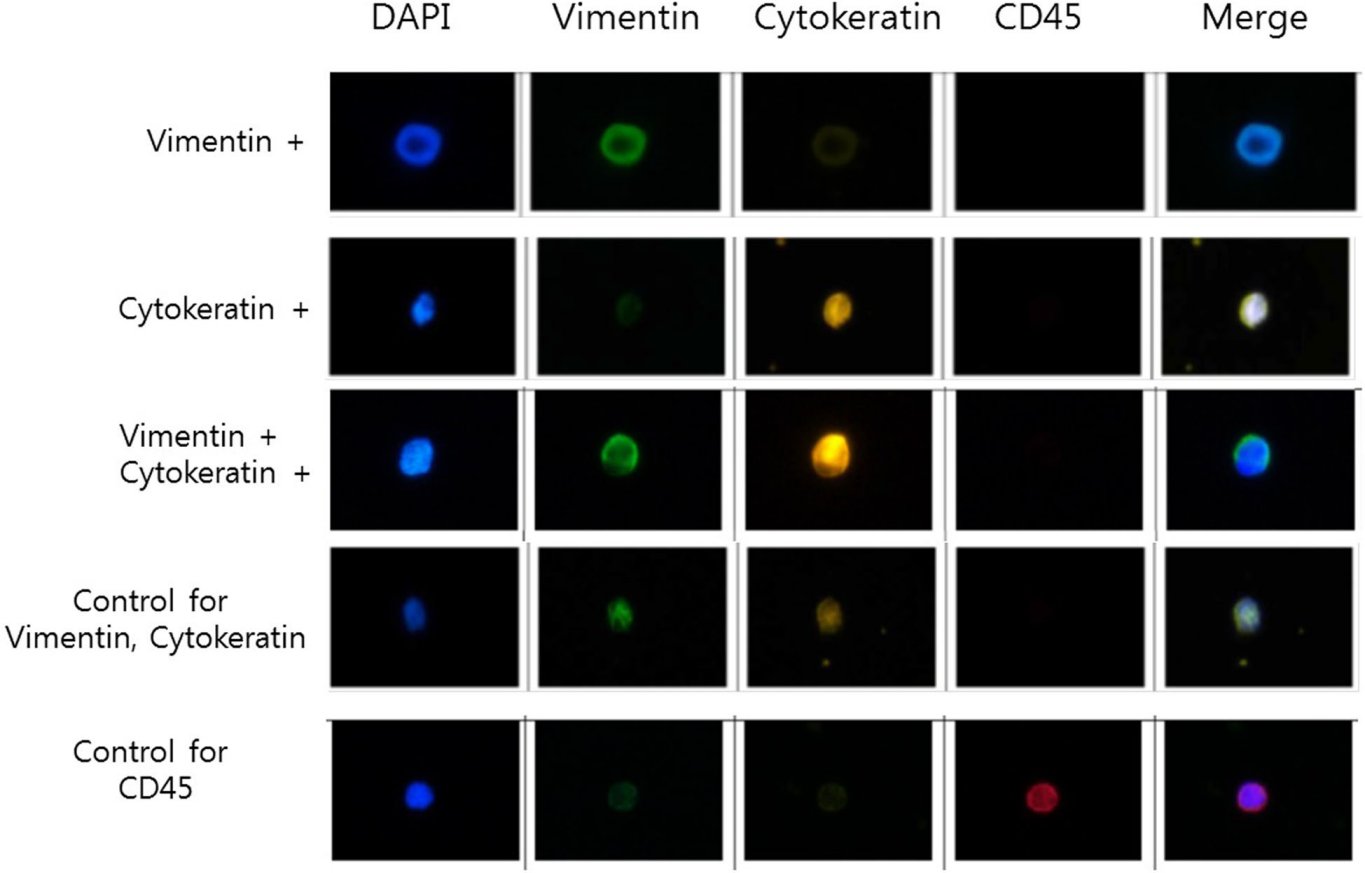
Representative immunostaining images showing circulating tumor cells (CTCs) with 4′,6-diamidino-2-phenylindole (DAPI), Vimentin, Cytokeratin, and CD45 positive

Blood samples were collected in acid citrate dextrose tubes from patients and processed within 4 h after sampling. All procedures of CTC isolation from blood were performed using Cell Isolation kit (#CIKW10; CytoGen, Inc., Seoul, Korea). Briefly, blood samples were incubated with an antibody complex against white blood cells (WBCs) and red blood cells at room temperature for 20 min and mixed with pre-activation buffer. WBC-depleted peripheral blood mononuclear cells (PBMC) were fractionated from whole blood by Ficoll density gradient centrifugation. The fraction of PBMC was diluted with a dilution buffer. Cell suspension was then filtered to isolate CTCs through a high-density microporous (HDM) chip. Enriched CTCs were fixed onto slides in 4% paraformaldehyde for 5 min at room temperature and kept at 4 °C until further processing. CTCs on slides were permeablized with 0.2% Triton X-100 in phosphate buffered saline (PBS) for 10 min at room temperature, blocked with 1% bovine serum albumin in PBS for 60 min, incubated with primary antibodies for 60 min, and then incubated with secondary antibody for 60 min. Primary antibodies used were rabbit anti-CD45 (Cell Signaling Technology), mouse anti-pan cytokeratin (Sigma), and mouse anti-vimentin Alexa Fluor® 488 conjugated (Cell Signaling Technology). Secondary antibodies used were goat anti-rabbit Alexa Fluor® 647 and goat anti-mouse Alexa Fluor® 546 (Thermo Fisher Scientific, Inc.). Slides were mounted using Fluoroshield with DAPI (ImmunoBioScience) and then detected using Nikon Eclipse Ti fluorescent microscope. Figure 1 shows representative fluorescent images of CTCs.

### Primary short-term culture of CTCs

CTCs retrieved on the HDM chip were washed with PBS and cultured in 6-well Costar® Ultra-Low Attachment plates (Costar®; Corning Korea Company, Ltd., Seoul, Korea) containing mesenchymal stem cell growth medium (MSCGMTM, human Mesenchymal Stem Cell Growth BulletKitTM Medium and Supplements; Lonza Group, Basel, Switzerland) at 37 °C in an atmosphere containing 5% CO_2_. Following 16–18 days of culture, cells were collected for exome sequencing analysis.

### Whole-exome sequencing

DNA extraction and library preparation were done as described previously [8]. To capture exonic DNA, Agilent SureSelect Human All Exome 50 Mb kit (Agilent, USA) was used. Paired-end 100 bp sequencing reads were generated on Illumina HiSeq 2500 platform (Illumina, USA) according to the manufacturer’s recommendation. Sequencing information is available in additional file 1.

### Somatic mutations

Sequencing reads in FASTQ files were aligned with human reference genomes (UCSC hg19) using BWA (Burrows-Wheeler alignment) aligner [9]. Local realignment and score recalibration of initial alignment results were done using Genome Analysis ToolKit [10]. Overall management and processing of sequencing data were done using SamTools and Picard [11]. To identify somatic mutations, sequencing data of primary tumors and CTC were compared with data of matched normal blood for each patient. Point mutations and short insertions/deletions (indels) were identified using MuTect and Indelocator, respectively [12]. ANNOVAR software was used to curate somatic mutations regarding resulting changes in amino acids [13].

## Results

### Patients

A total of 20 patients with non-metastatic bladder cancer were enrolled in this study. Clinical characteristics of these 20 patients with counts and immunophenotypes of CTCs are shown in Table 1. Regarding pathological staging, there were 3 patients with Ta, 4 patients with T1, 4 patients with T2, 7 patients with T3, and 2 patients with T4. CTC counts at baseline were reported for all cohorts. There was a general tendency of increased number of CTC with tumor stage (pT1 to pT4). However, the immunophenotype of captured CTCs showed a wide distribution even for those with the same stage.

**Table 1:** Clinical characteristics and immunophenotype of initial captured CTCs from bladder-cancer patients

				Immunophenotyping & Enumeration (5 ml)			
Subject number	Pathologic T stage	LVI	Vein invasion	Vimentin (+), CK (−)	Vimentin (−), CK (+)	Vimentin (+), CK (+)	Total CTC
TCC14	T4a	Pos	Neg	1	17	0	18
TCC16	T1	Neg	Neg	1	7	1	9
TCC17	T3a	Pos	Neg	1	5	2	8
TCC18	T3a	Neg	Neg	0	17	1	18
TCC20	T2b	Pos	Pos	6	1	21	28
TCC21	T3a	Pos	Pos	0	0	15	15
TCC27	T3a	Neg	Neg	0	11	0	11
TCC28	T2b	Pos	Neg	0	12	5	17
TCC31	T2a	Pos	Pos	9	1	4	14
TCC32	T3a	Pos	Pos	3	11	18	32
TCC37	Ta	Neg	Neg	1	1	0	2
TCC38	T3a	Neg	Neg	1	12	17	30
TCC39	T1	Neg	Neg	0	4	4	8
TCC40	Ta	Neg	Neg	2	1	4	7
TCC41	Ta	Neg	Neg	0	1	2	3
TCC43	T1	Neg	Neg	1	2	7	10
TCC44	T1	Neg	Neg	1	2	5	8
TCC45	T3a	Pos	Pos	0	19	8	27
TCC46	T2a	Neg	Neg	4	2	4	10
TCC47	T2a	Neg	Neg	2	1	7	10

*LVI* Lymphovascular invasion

### Somatic mutations of primary bladder cancers and CTCs

**Fig. 2:**
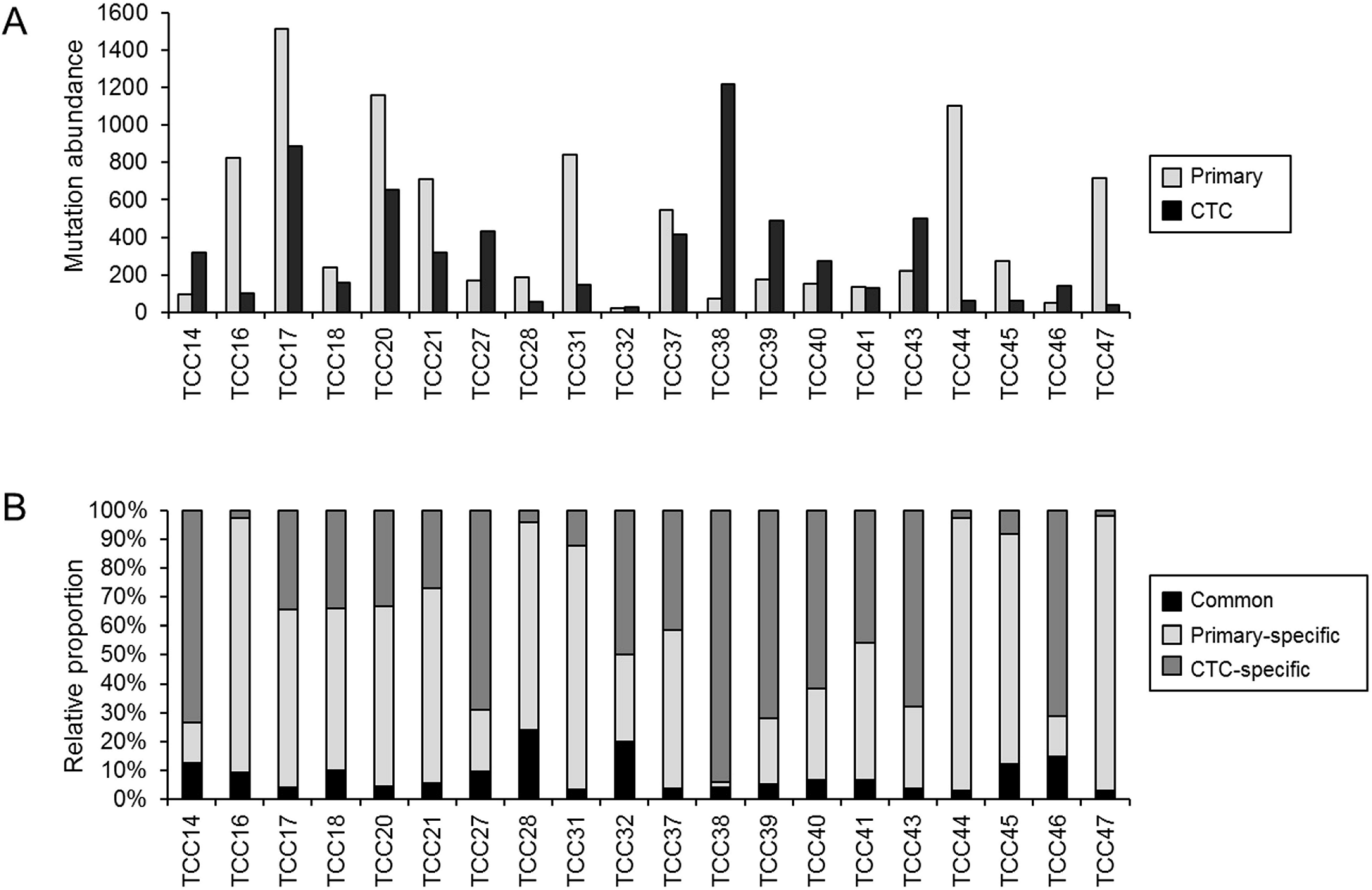
Mutation abundance of primary tumors and CTC (circulating tumor cells) genomes. (**a**) Mutation abundance of primary tumors and their matched CTC genomes for 20 cases of UBC. (**b**) Relative proportion of common mutations (i.e., those commonly observed both for primary and CTC genomes) and region-specific mutations in given cases

Somatic mutations were identified from primary tumor and CTC genomes by comparing them with matched normal genomes. For 20 pairs of primary and CTC genomes, we identified a total of 14,864 exonic mutations **(**Additional file 2**)**. Mutation abundance was highly variable across tumors, i.e., 20–1515 exonic mutations (median of 231) for primary tumors and 28–1217 exonic mutations (median of 217) for CTC genomes (Fig. 2a). Mutation abundances of primary tumors and CTC genomes were not significantly correlated (*r* = 0.16, *P* = 0.499), suggesting that mutation abundance of primary tumors might not have been accurately inferred from CTC mutation profiles. Next, we classified mutations into common and primary−/CTC-specific mutations. Common mutations were those observed both in the primary and CTC genomes while primary and CTC-specific mutations were those observed only in the primary and CTC genomes, respectively. Relative proportions of common and primary−/CTC-specific mutations in 20 bladder cancers are shown in Fig. 2b. We identified 8 to 89 common mutations comprising 2.7 to 23.9% of mutations in a given case across 20 cases. The low level of mutation concordance and low correlation of mutation abundance between the primary and CTC genomes suggest that CTC might have diverged from primary tumors in early evolutionary stages and undergone distinct genome evolution.

### Mutation features with respect to regional categories

**Fig. 3:**
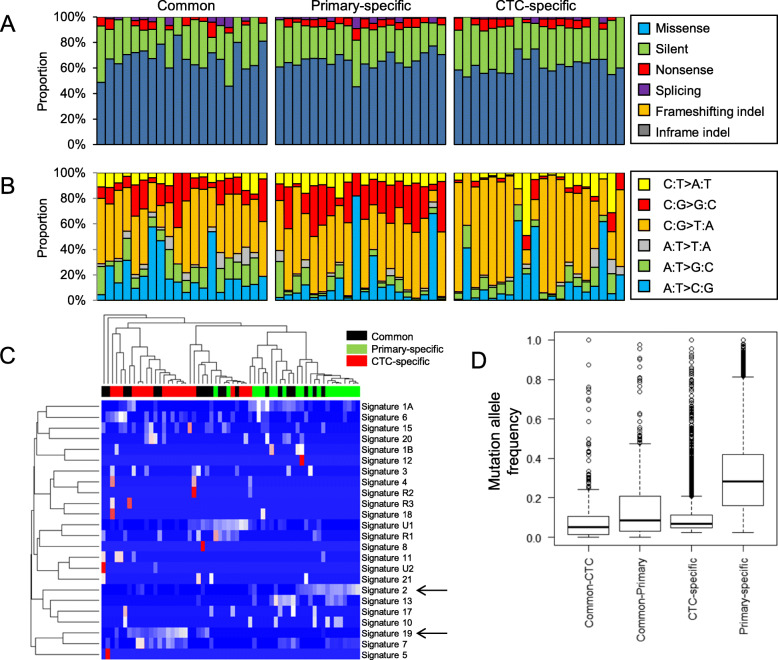
Three mutation categories and mutation analysis. (**a**) Consequences of somatic mutations on encoded peptide sequences are categorized and relative proportions are shown across three regional mutation categories (common, primary-specific, and CTC-specific). (**b**) Similarly shown for six mutation spectra. (**c**) Mutation signature levels estimated across mutation categories are shown in a heatmap with a dendrogram. Two arrows indicate signatures of 2 and 13. (**d**). Mutation allele frequencies of mutations are shown as clonality measures

Common mutations as well as primary- and CTC-specific mutations were examined for functional consequence of somatic mutations on encoded peptides (e.g., missense and silent mutations) (Fig. 3a). We observed that ratios of missense mutations to silent mutations (NS/S ratios) were similar across regional categories (1.1–6.0, 1.2–4.2, and 1.1–3.7 for common, primary-specific, and CTC-specific mutations, respectively). The comparable level of NS/S ratios across different mutation categories and the comparable mutation abundance of primary tumors and CTC genomes indicate that mutations identified in CTC genomes are somatic mutations arising from tumors instead of germline variants [14]. However, mutation spectra analysis showed that CTC-specific mutations were distinct from common or primary-specific mutations (Fig. 3b). A depletion of C-to-G transversions and an overrepresentation of C-to-T transitions were observed specifically for CTC-specific mutations compared to common and primary-specific mutations. We further explored mutation signatures across the three mutation categories (Fig. 3c). Hierarchical clustering of signature levels revealed two clusters representing primary- and CTC-specific mutations were relatively enriched with mutation signatures 2 and 19, respectively (arrows in Fig. 3c). Mutation signature 2 representing the activity of endogenous APOBEC cytidine deaminase has been associated with UBC in TCGA analysis [15, 16]. Mutation signature 19 is characterized by C-to-G transversions, but distinct from C-to-G transversions occurring on CpG dinucleotide contexts (e.g., signature 1A and 1B representing spontaneous deamination of methylated CpG dinucleotides) [17]. This signature has been observed in pilocytic astrocytomas whose mutation etiology is largely known. Mutation spectra and signature analysis results suggest that mutational forces that have been operative in the generation of primary- and CTC-specific mutations are not identical, further highlighting that CTC genomes might have evolved separately from primary tumors. Clonality analysis results of mutations in terms of mutant allele frequencies are shown in Fig. 3d. Mutations in primary and CTC genomes were segregated into common and region-specific mutation. Of interests, region-specific mutations showed higher level of mutant allele frequencies compared to region-common mutations. These results suggest that common mutations observed in both primary tumors and CTCs might not represent clonal mutations and that the mutational subclonal architecture of two genomes might have been subjected to selection events.

### Cancer-related genes

To identify functional driver mutations in each regional category, we first examined cancer-related genes (i.e., Cancer Census Genes [18]) that occurred in at least three cases in our cohort (Table 2). In region-common mutations, missense mutations of *KMT2C* encoding MLL3 were frequently observed (5 cases), suggesting that mutations on this epigenetic regulator might represent early genomic alterations in the development of bladder cancer. *KMT2C* gene has been frequently observed to be mutated in muscle invasive bladder cancer [19]. Consistently, *KMT2C* mutations were also frequently observed in CTC- and primary-specific mutations including one and two nonsense mutations, respectively. Frequent mutations on other epigenetic modifiers in CTC- and primary-specific mutations were not identical in that CTC-specific mutations were observed on *ASXL1* and *TET1* while primary-specific mutations were common in *KMT2D*, *KDM6A*, and *ARID1A*. Pathway-level convergent mutations targeting different context of epigenetic regulators highlight the importance of epigenetic dysregulation in the pathogenesis of bladder cancers. Of note, CTC-specific frequent mutations included those associated with hematologic malignancies such as *ASXL1* [20] *and SETBP1* [21] suggestive of environmental impact on the acquisition of mutation. Previously reported mutations that were frequent in muscle invasive bladder cancers such as *TP53*, *PIK3CA*, *RB1*, *EP300*, and *FGFR3* were observed in primary-specific mutations.

**Table 2:**
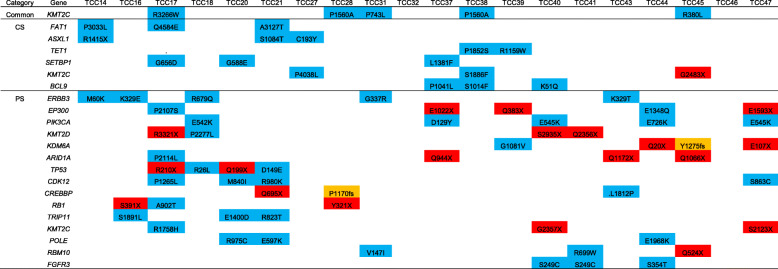
Mutations on cancer-related genes

## Discussion

In this study, we observed that the concordance rate of somatic mutations between CTC and corresponding primary tumor genomes was relatively low, suggesting that CTC genomes might have undergone distinct evolutionary pathway compared to primary tumors. Supporting this, CTC-specific mutations are distinct from common or primary-specific mutations in terms of sequence compositions (i.e., mutation signatures) and clonality measures such as allele frequencies, suggesting that mutational forces for CTC genomes might be distinct from those associated with primary tumors. In addition, cancer-related genes affected by CTC- and primary-specific mutations were also different from each other.

Research on clinical relevance of ‘liquid biopsy’ has been recently highlighted. CTC may be used as a substitute for tissue biopsy to evaluate drug responsiveness and predict an optimal therapy [22, 23]. CTC-driven genomic or transcriptomic findings may provide valuable information for predicting patients’ prognosis and further guide personalized treatment decision-making in bladder cancer. Until now, primary tumor specimens have served as major cellular sources to obtain genetic information. Some studies have compared genomic or transcriptomic profiles and reported a substantial level of heterogeneity between primary and matched metastatic lesions of given individual [24, 25]. Our study performed analyses of genomic profiles of cultured CTCs and corresponding primary tumor tissue. The current study presented some meaningful results. As demonstrated here, genomic profiling of primary tumors and CTCs is very different in other studies. Reports for colorectal cancer showed similar results. For example, Lyberopoulou et al. [26] have observed 52 patients with colorectal cancer and found discordance between primary tumor and CTCs for *KRAS*, *BRAF*, *CD133*, re3130, and Plastin3 rs6643869. This discordance was confirmed by Kondo et al. [27] and a recent meta-analysis including nine studies and 244 patients [28]. We expect that mutational heterogeneity of CTCs and corresponding primary bladder tumor may recapitulate the relationship between primary and metastatic lesions given that CTCs are major sources for distant metastases. To cope with metastasis and disease progression, genetic information from CTCs might be complementary for longitudinal management of bladder cancer.

To explain the discordance between CTCs and corresponding primary tumor, we need to pay attention to intra-tumor heterogeneity (ITH) and CTCs heterogeneity. Modeling genomic or mutational diversity between regional biopsies in a given individual using a tree structure of tumor growth has been proposed as a ‘trunk-branch model’ [29]. The trunk and branch/leaf in the tree represent founding ubiquitous driver mutations present in every tumor subclone and region and regionally heterogeneous mutations that are not present in every tumor cell or tumor region, respectively [29]. The number of ubiquitous and heterogeneous genetic events in the tumor can depend on the length of the trunk and size of the branch of the phylogenetic trees inferred from mutation profiles and their regional distribution. Thus, as disease progresses, in addition to mutations detected in primary tumors, CTC genomes can acquire mutations in a similar evolutionary process where minority subclones emerge from pre-existing clones. Tumor cells can shed from different and many other tumor sites. Hence, mutation profiles of CTC genomes may be substantially different from those of primary tumors as composite of genetically distinct tumor subject to ITH [30]. Thus, single site tumor biopsy may underestimate the clonal landscape of the overall tumor burden.

Among mutations commonly observed in both CTCs and corresponding primary tumors, *KMT2C* mutations were detected in five patients. All these five patients (patients TCC 17,28,31,38,45) were shown to be pathologically T2 or higher stage (muscle invasive bladder cancer). *KMT2C* gene has been frequently observed to be mutated in muscle invasive bladder cancers [19]. On the contrary, *KMT2C* mutations in CTCs were not observed in patients with T1 or Ta disease. In clinical practice for bladder cancer, T2 disease has a great clinical significance in decision-making for treatment. Although it requires further supporting evidence, the correlation of disease stages and certain mutations such as *KMT2C* is one of major applications in using CTCs for clinical practice.

To the best of our knowledge, this is the first study to conduct mutation-based comparison between CTCs and primary corresponding tumor mutations using whole-exome sequencing in bladder cancer. Although a number of studies have used high-throughput sequencing to characterize CTCs for bladder cancers [31], their results are only for CTC, not amenable for comparison between CTC and primary tumors. In addition, the present study suggests that the application of sequencing or CTCs could provide a potential clinical role to investigate biologic targets by peripheral blood sampling. Genetic characteristics of CTCs and corresponding primary tumor were shown to differ. Thus, identifying only genetic characteristics of primary tumors is not enough to cope with the disease’s progression. Clearly, CTCs are expected to play an important role. The need for genetic analysis using liquid biopsy such as CTCs can also play a role in handling bladder cancer in addition to convenience of sampling in liquid biopsy. Moreover, recent studies addressed the genomic alterations associated with distant metastases, e.g., *ESR1* mutations and *MYC*, *YAP1* amplifications enriched in metastatic breast cancer and lung cancer genomes, respectively [32, 33]. These studies also highlight the difference in terms of driver mutations. Given the CTCs represent the cells that have escaped the physical constrains of primary tissues, the comparison of somatic mutations and other genomic alterations between primary and CTC genomes would provide clues on the genetic drivers of distant metastases.

One limitation of this study is an incomplete establishment of CTC culture. Despite the importance of CTCs in prognosis of cancer patients, the clinical utility of CTCs was limited by its rareness in blood (one CTC in a billion normal blood cells) [34]. For this reason, expanding CTCs to large numbers is encouraged for performing CTC genotyping and phenotyping despite there are concerns that in vitro culture of CTCs may change characteristics of bona fide CTCs. It can be questionable whether cultured CTCs originating from primary cancer could retain their original identification. To verify if cultured CTCs could retain their original identification of primary bladder cancer, we have previously verified that cultured CTCs could retain their original identification of primary bladder cancer by applying FISH method although further studies are needed [35]. In addition, bladder cancer tissue specimens were obtained by TURBT in our study. Although tissue specimens were acquired with sufficient margins, which were removed before the tissue preparation, tissue and DNA damage may have occurred during TURBT. In order to minimize or avoid these concerns, samples were obtained with TURBT preferably thick and large as much as possible. Then, samples were frozen and stored after removing the boundary parts that are suspected of being damaged.

## Conclusion

In this mutation-based comparison study, we observed a low concordance level of mutations between genomes of CTCs and primary corresponding bladder cancers, suggesting that CTC genomes might have undergone distinct evolutionary pathway compared to primary tumors. The current study presented some meaningful results to be taken into account when evaluating clinical utility of circulating tumor cells for treatments and follow-up of bladder cancers.

## Supplementary Information

**Additional file 1.**

**Additional file 2.**

## Data Availability

The datasets analyzed during the current study would be available from the corresponding author on reasonable request.

## Abbreviations

CTCs: Circulating tumor cells
TURBT: Transurethral resection of bladder tumor
ITH: Intra-tumor heterogeneity
